# Whole-Mount MeFISH: A Novel Technique for Simultaneous Visualization of Specific DNA Methylation and Protein/RNA Expression

**DOI:** 10.1371/journal.pone.0095750

**Published:** 2014-04-22

**Authors:** Hirosuke Shiura, Akimitsu Okamoto, Hiroyuki Sasaki, Kuniya Abe

**Affiliations:** 1 Technology & Development Team for Mammalian Genome Dynamics, RIKEN BioResource Center, Ibaraki, Japan; 2 Research Center for Advanced Science and Technology, The University of Tokyo, Tokyo, Japan; 3 Division of Epigenomics and Development, Medical Institute of Bioregulation, and Epigenome Network Research Center, Kyushu University, Fukuoka, Japan; 4 Graduate School of Life and Environmental Sciences, University of Tsukuba, Ibaraki, Japan; University of Science and Technology of China, China

## Abstract

To understand the spatiotemporal changes in cellular status that occur during embryonic development, it is desirable to detect simultaneously the expression of genes, proteins, and epigenetic modifications in individual embryonic cells. A technique termed methylation-specific fluorescence *in situ* hybridization (MeFISH) was developed recently that can visualize the methylation status of specific DNA sequences in cells fixed on a glass slide. Here, we adapted this glass slide-based MeFISH to the study of intact embryos, and established a method called whole-mount MeFISH. This method can be applied to any DNA sequences in theory and, as a proof-of-concept experiment, we examined the DNA methylation status of satellite repeats in developing mouse primordial germ cells, in which global DNA demethylation is known to take place, and obtained a result that was consistent with previous findings, thus validating the MeFISH method. We also succeeded in combining whole-mount MeFISH with immunostaining or RNA fluorescence *in situ* hybridization (RNA-FISH) techniques by adopting steps to retain signals of RNA-FISH or immunostaining after harsh denaturation step of MeFISH. The combined methods enabled the simultaneous visualization of DNA methylation and protein or RNA expression at single-cell resolution without destroying embryonic and nuclear structures. This whole-mount MeFISH technique should facilitate the study of the dynamics of DNA methylation status during embryonic development with unprecedented resolution.

## Introduction

Various imaging techniques have been applied successfully to the analysis of cellular status. For example, the RNA fluorescence *in situ* hybridization (RNA-FISH) method is used to detect nascent transcripts from a particular gene, and the expression and localization of proteins in cells or tissues can be visualized by immunocytochemistry/histochemistry. Despite its importance, methods of visualization of the epigenetic status at specific loci in individual cells have not been pursued extensively. DNA methylation is one of the most important epigenetic modifications that regulate gene expression in many organisms [1]. The global distribution of DNA methylation in the nucleus of each cell can be visualized by immunocytochemistry using an antibody against 5-methylcytosine [2]. Global DNA methylation patterns and/or heterochromatin morphologies in living cells can be visualized using a fluorescent protein fused with a methylated DNA-binding motif [3]. However, these two methods cannot be used to address the methylation status of specific DNA sequences. The methylation status of specific DNA sequences is usually analyzed by bisulfite sequencing [4]. However, this technique is not suited to *in situ* analysis. Tanaka et al. [5] reported a novel method to detect methylated cytosines at specific DNA sequences. This method is based on a principle that is totally different from the widely used bisulfite conversion, and uses interstrand complexes formed by osmium and nucleic acids (ICON) probe technology. The ICON probe contains a bipyridine-attached adenine derivative, which has much higher binding ability to 5-methylcytosine (5 mC) as well as 5-hydroxymethylcytosine (5 hmC) than does the unmodified cytosine at the complementary position to the bipyridine-attached adenine in target DNA after treatment with osmium [5], [6]. Taking advantage of the tight binding of the ICON probe to 5 mC or 5 hmC, one can assay CpG methylation of specific sequences using a quantitative polymerase chain reaction (PCR) [5]. Moreover, because of the strong binding of the ICON probe to 5 mC, the probe should be applicable to FISH analysis for the assessment of DNA methylation. Recently, Li et al. [7] developed a novel method, methylation-specific fluorescence *in situ* hybridization (MeFISH), for the detection of the DNA methylation status at specific sequences in individual cells (see Figure S1 for an overview of MeFISH). Using this method, the authors succeeded in visualizing the DNA methylation statuses of the satellite repeat sequences in spreads of nuclei/chromosomes or frozen sections on slides [7]. To our knowledge, this method is the first to show the methylation status of specific DNA sequences *in situ*. As this MeFISH protocol is adapted for specimens that have been prepared on a glass slide, it has the limitation of not being suited to the imaging of whole embryos or embryonic tissues.

Here, we extended the utility of the MeFISH technology to the imaging of intact early embryos or small embryonic tissues using the sample preparation method established previously by our group [8]. This whole-mount MeFISH approach has enabled the observation of cells in intact embryos and of nuclear structures within these cells. To understand the importance of the spatiotemporal changes in cellular and epigenetic statuses that occur during embryonic development, it is desirable to observe embryos without destroying their morphological integrity. Moreover, we developed methods that combine whole-mount MeFISH with immunofluorescence or RNA-FISH. The combined analyses allowed the three-dimensional visualization of the DNA methylation status of specific sequences and the protein localization or RNA expression in cells of intact mouse embryos. In particular, to our knowledge, the combination of whole-mount MeFISH and RNA-FISH is the first technique that can be used for the simultaneous detection of DNA methylation at specific loci and of RNA expression at the single-cell resolution. As a proof-of concept experiment, here, we applied this technique to examine the methylation status of satellite repeat sequences in the genome of developing mouse primordial germ cells (PGCs), in which epigenetic reprogramming is known to take place [8]–[10]. The hypomethylated state of the satellite repeats in the PGC genome revealed by MeFISH was consistent with the results obtained by bisulfite sequencing [11]. Therefore, this whole-mount MeFISH technique should facilitate the understanding of the epigenetic basis of cellular dynamics during embryonic development.

## Materials and Methods

### ICON Probe

The ICON probe for mouse major satellite repeats was the same as that used by Li et al. [7]. The probe was labeled with biotin using biotinamidohexanoyl-6-aminohexanoic acid *N*-hydroxysuccinimide ester (Sigma-Aldrich, St. Louis, Missouri, USA). The sequence of the probe was 5′–catccacttgacgacttgaaaatgacBaaatcactaaaaaacgtg–3′ (B indicates a bipyridine-attached adenine derivative).

### Mice

The *Blimp1-mRFP* transgenic mouse line established by Sugimoto and Abe [8] was maintained by breeding with C57BL/6JxDBA/2 F1 (BDF1) mice. The mouse strain, B6.Cg-Tg(Blimp1-RFP)1Rbrc, was obtained from the Experimental Animal Division of the RIKEN BioResource Center. Embryos of 6.5 days post coitum (dpc) were recovered from BDF1 mothers crossed with Blimp1-mRFP males for combined MeFISH/immunostaining analysis. For MeFISH or combined analysis of MeFISH/RNA-FISH, male mice with a mixed background of C57BL/6J and DBA/2 were crossed to BDF1 female to obtain 6.5 dpc embryos and 12.5 dpc embryos from which genital ridges were recovered. Noon of the day when vaginal plugs were detected was set as 0.5 dpc. All animal protocols were approved by the Institutional Animal Experiment Committee of RIKEN Tsukuba Institute.

### Permeabilization and Fixation of Embryos and Embryonic Tissues

Before conducting MeFISH, immunostaining or RNA-FISH, whole embryos or embryonic tissues were subjected to permeabilization and fixation as described previously [8]. Permeabilization with detergent was conducted to ensure efficient ICON probe penetration into nuclei. Condition of permeabilization should be determined empirically for each sample. For example, 6.5 dpc embryos were incubated in 0.5% Triton X-100 in PBS for 2 min on ice, while genital ridges from 12.5 dpc embryos were treated in 0.5% Triton X-100 for 15 min on ice. After permeabilization, samples were fixed with 4% paraformaldehyde in PBS with 0.1% Triton X-100 (PBST) for 10 min at room temperature. Samples were washed three times with PBST for 5 min at room temperature. For genital ridges, an additional permeabilization step with 10 mg/ml Proteinase K in PBST for 5 min at 37°C was added, and the samples were refixed with 4% paraformaldehyde in PBST for 15 min. After these treatments, samples were used for subsequent experiments, i.e. MeFISH, immunostaining, or RNA-FISH.

As permeabilization is performed before fixation in the MeFISH protocol, leakage of target antigens from the cell samples may occur. Therefore, it is important to establish appropriate permeabilization conditions when immunocytochemistry is combined with MeFISH. For immunostaining of PGC precursors of 6.5 dpc embryos with an anti-RFP antibody, we were able to retain the immunofluorescence signal in the PGC precursors using the conditions described above.

### Whole-mount MeFISH

Samples processed as above were subjected to whole-mount MeFISH. Samples were incubated sequentially in 2xSSC with 0.1% Triton X-100 for 10 min, 2xSSC and 25% formamide with 0.1% Triton X-100 for 10 min, and 2xSSC and 50% formamide with 0.1% Triton X-100 for 10 min twice. After prehybridization of the samples in hybridization buffer (2xSSC, 2 mg/ml bovine serum albumin (BSA), 0.1% Triton X-100, and 50% formamide) for 20 min, the samples were placed in a 1.5 ml plastic tube containing 30 µl of the hybridization buffer containing the major satellite ICON probe (0.1 ng/µl). After denaturation of genomic DNA by heating the samples at 98°C for 5 min, hybridization was performed overnight at room temperature (approximately 25°C). The samples were then washed three times in 2xSSC with 0.1% Triton X-100 at room temperature for 5 min, to remove unhybridized probe. To cross-link ICON probes with methylated target sequences, the samples were incubated in 100 µl of cross-link solution at 30°C for 10 min. The cross-link solution was made by mixing an equal amount of 25 mM K_2_OsO_4_•2H_2_O and a Tris-HCl solution (100 mM Tris-HCl (pH 7.4), 1 mM EDTA, 2 M NaCl, and 0.1% Triton X-100). Probes that were not cross-linked were removed by denaturation at 80°C for 15 min in a solution containing 90% formamide, 2xSSC and 0.1% Triton X-100, followed by washing in PBST. The biotinylated ICON probe was detected using the Tyramide Signal Amplification (TSA) Kit (Life Technologies Corporation, Carlsbad, CA USA). After incubation of the samples in 3% BSA in PBST, endogenous peroxidase activity was quenched by treatment with 0.6% hydrogen peroxide for 10 min. After several washes in PBST, the samples were reacted with a streptavidin–HRP conjugate in 3% BSA in PBST for 30 min. The samples were washed three times in PBST for 5 min at 37°C, followed by incubation with a fluorescence-labeled tyramide solution (Alexa Fluor 488 or 546) with 0.0015% hydrogen peroxide. The tyramide solution used in this step had a different fluorescence wavelength from the one used in immunostaining or RNA-FISH. The samples were washed several times in PBST, and nuclear DNA was stained with TO-PRO-3 (Life Technologies Corporation, Carlsbad, CA USA). Samples were imaged with a confocal fluorescence microscope (LSM510; Carl Zeiss, Jena, Germany).

### Immunostaining Combined with MeFISH

The permeabilization and fixation of the samples were performed as described above. After incubation in blocking buffer (1% BSA in PBST) for 60 min, the samples were reacted with the primary antibody (for mRFP, 1∶1000 rabbit polyclonal anti-RFP (MBL, Nagoya, Japan, PM005); for OCT4, 1∶300 goat polyclonal anti-Oct-3/4 (N-19) (Santa Cruz Biotechnology, Santa Cruz, California, USA, sc-8628)) in blocking buffer at 4°C overnight. When combining immunostaining with MeFISH, we used the TSA Kit for the detection of the target protein. Briefly, after PBST wash and quenching of endogenous peroxidase activity with 0.6% hydrogen peroxide, the samples were incubated with the secondary antibody conjugated with horseradish peroxidase (HRP) (for mRFP, goat anti-rabbit IgG conjugated with HRP (Life Technologies Corporation, Carlsbad, CA USA, G21234); for OCT4, rabbit anti-goat IgG conjugated with HRP (Life Technologies Corporation, Carlsbad, CA USA, R21459)) in blocking buffer at 4°C overnight. After rinsing with PBST, the samples were incubated with a fluorescence-labeled tyramide solution (Alexa Fluor 488 or 546) with 0.0015% hydrogen peroxide. After washing several times in PBST, the samples were then subjected to whole-mount MeFISH experiment.

### RNA-FISH Combined with MeFISH

A strand-specific DNA probe for the detection of the *Xist* RNA was prepared as follows. A single-stranded RNA corresponding to a part of the *Xist* cDNA (1986–5786 bp; NR_001463) was synthesized by *in vitro* transcription using T7 RNA polymerase (Roche, Mannheim, Germany, 881767). Using the *in vitro*-transcribed RNA as a template, a biotinylated single-stranded DNA probe was generated by random-primed reverse transcription using SuperScript III reverse transcriptase (Life Technologies Corporation, Carlsbad, CA USA, 18080-044). Samples subjected to RNA-FISH were processed using the same procedures as those described in the whole-mount MeFISH protocol, up to the prehybridization step. The biotinylated *Xist* probe dissolved in hybridization buffer was denatured by heating at 70°C for 10 min and the samples were added into the hybridization mixture. Hybridization was performed at 42°C overnight and the samples were washed twice with a solution containing 2xSSC, 50% formamide and 0.1% Triton X-100 for 5 min at 42°C, and twice with 2xSSC containing 0.1% Triton X-100 for 5 min at 42°C. For the fluorescence labeling of the probes and stabilization of the signals, TSA was performed as described above. After TSA procedures, the Endogenous Biotin-Blocking Kit (Life Technologies Corporation, Carlsbad, CA USA, E21390) was used to block unreacted biotin in the probe with the streptavidin–HRP conjugate. The samples were then used in whole-mount MeFISH experiments.

## Results

**Figure 1:**
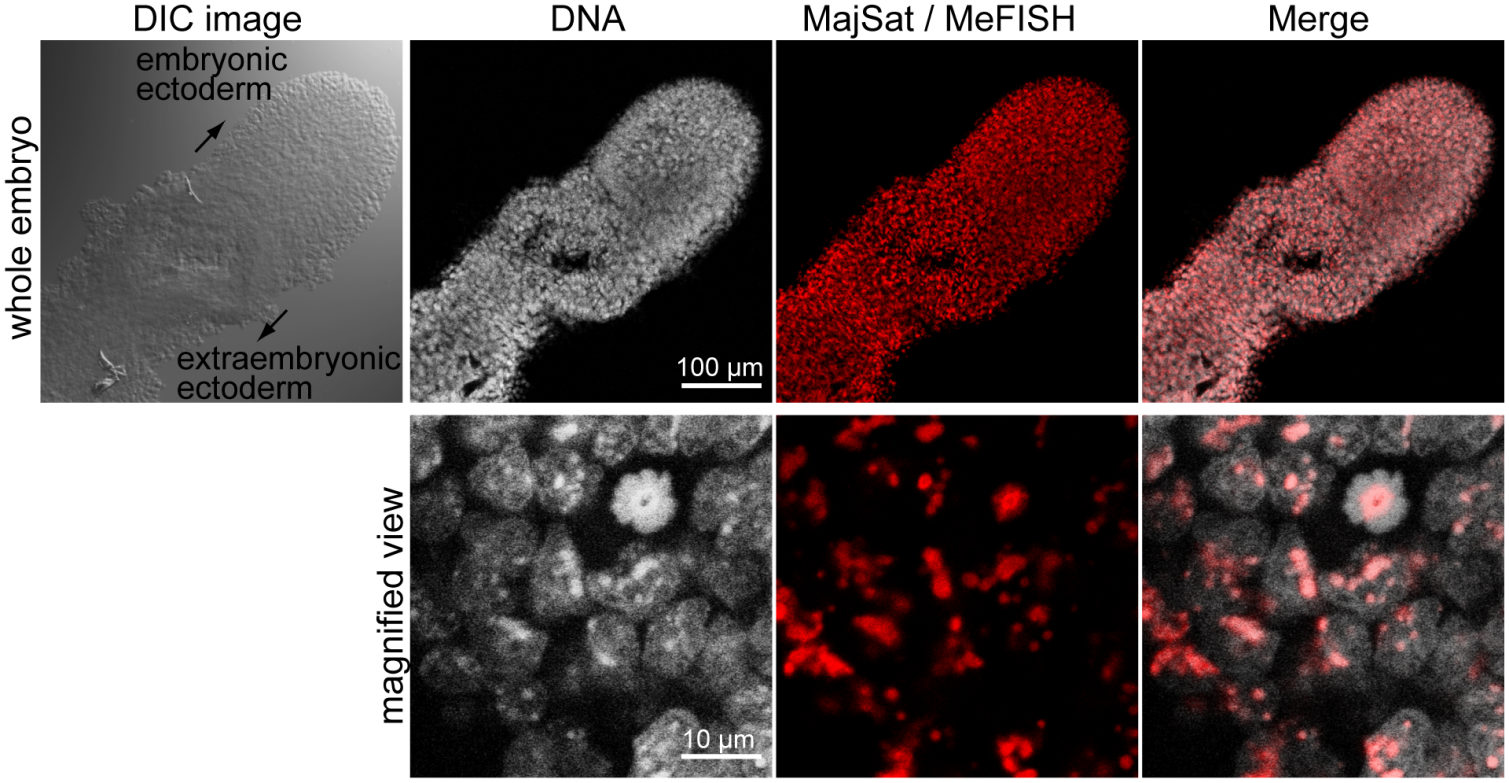
Establishment of the whole-mount MeFISH method. Mouse embryos at 6.5-mount MeFISH for major satellite sequences (red). Nuclear DNA was stained with TO-PRO-3 (white). The lower panels are magnified views of the upper images.

The original MeFISH protocol was created for the analysis of either dissociated cells or frozen sections adhered to a glass slide [7]. The hypotonic treatment and methanol/acetic acid fixation used in the above study would disrupt morphological integrity of embryos and nuclear structure of each embryonic cell. To perform MeFISH analysis in early embryos or small embryonic tissues of mice without disrupting embryonic structures, we used the sample preparation procedures for the whole-mount RNA-FISH method that were established previously by our group [8]. We used 4% paraformaldehyde as a fixative to retain the morphologies of embryos and the three-dimensional structures of nuclei after fixation. In addition, a sample permeabilization step using Triton X-100 detergent was included before the fixation for efficient probe penetration into nuclei. Using an ICON probe for major satellite repeat sequences, we performed a whole-mount MeFISH technique using intact mouse embryos at 6.5 dpc (Figure 1). It is known that the mouse major satellite repeats located in pericentromeric regions are heavily methylated [11] and can thus be densely stained with 4′, 6-diamidino-2-phenylindole (DAPI), TO-PRO-3, and other nuclear-staining reagents [12], [13]. Figure 1 shows that MeFISH signals on the major satellite sequences were detected in regions that overlapped with TO-PRO-3-dense staining in each cell, indicating that the whole-mount MeFISH method detected the methylation of the major satellite repeats, as expected.

**Figure 2:**
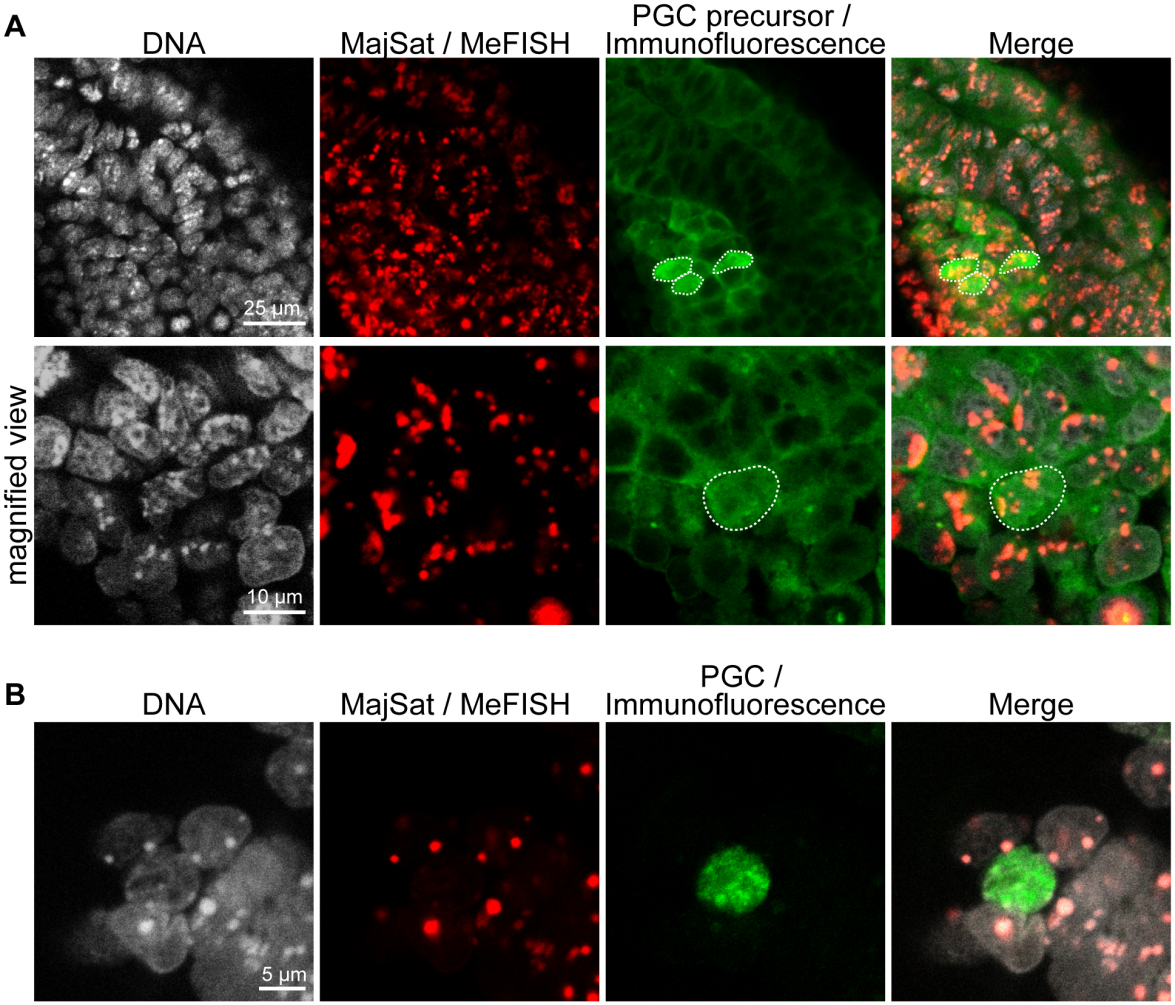
Whole-mount MeFISH in combination with immunofluorescence. A) Whole-mount MeFISH for major satellite sequences (red) in PGC precursors marked by Blimp1-mRFP expression (green) in a 6.5 dpc embryo. The mRFP-positive PGC precursors are circled with white dotted lines. The lower panels are magnified views of the upper images. B) MeFISH for major satellite sequences (red) in PGCs marked by anti-OCT4 immunostaining (green) in a genital ridge at 12.5 dpc. The nuclei of OCT4-positive PGCs show almost no, or only very faint, MeFISH signals. Nuclear DNA was stained with TO-PRO-3 (white).

Next, we attempted to assess the methylation status of the satellite sequences in precursors of mouse PGCs using a combination of whole-mount MeFISH and immunocytochemistry. Global loss of DNA methylation in PGCs is observed at around 8.0 dpc [14], although the global DNA methylation status in PGC precursors has not been investigated. Precursors of PGCs can be marked by the expression of *Blimp1*/*Prdm1* in mouse 6.5 dpc embryos [15]. We used the *Blimp1*-*mRFP* transgenic mice, in which PGC precursors can be detected by the expression of monomeric red fluorescent protein (mRFP) [8]. We stained a whole 6.5 dpc embryo with an anti-RFP antibody to identify PGC precursors. After immunostaining, MeFISH was performed using the major satellite probe. As shown in Figure 2A, mRFP-positive PGC precursors were detected at proximal, posterior parts of the epiblast. The MeFISH signals overlapped with TO-PRO-3-dense regions in all cells, including PGC precursors, and the intensity of the hybridization signals seemed to be unaltered between the PGC precursors and the surrounding somatic cells. This result suggests that the major satellite sequences in the genome of PGC precursors are methylated or modified by 5-hydroxymethylation. This combined analysis allowed the visualization of the methylation status of the target DNA sequences in a specific cell lineage in an intact embryo.

To address whether these techniques can be applied to larger samples, such as pieces of embryonic tissues or organs from embryos at later developmental stages, and to determine if differences in the DNA methylation levels between distinct cell lineages can be detected, we performed a MeFISH analysis of both somatic cells and PGCs in the genital ridge of 12.5 dpc embryos; PGCs within the genital ridges of this stage should be globally demethylated [14], whereas the somatic cells should show hypermethylation at the major satellite repeat sequences [11]. As shown in Figure 2B, almost no, or only very faint, MeFISH signals were detected in the PGCs marked by the anti-OCT4 antibody. Movies S1 and S2 demonstrate the spatial locations of PGCs within the genital ridges. The result of MeFISH, i.e. faint signals for the satellite repeats, is consistent with the results of bisulfite sequencing analyses of the major satellite repeats in PGCs [11], [16]. On the other hand, the somatic cells that surround the PGCs showed strong signals for the highly methylated major satellite repeat sequences. The signals detected in somatic cells served as a positive control for the MeFISH procedure, ensuring the great reduction of DNA methylation at the major satellite repeats in PGCs. The results of this experiment suggest that the combination of whole-mount MeFISH and immunofluorescence staining allows the identification of differences in the DNA methylation status of distinct cell lineages that constitute three-dimensional embryonic structures without loss of information regarding their spatial location.

**Figure 3:**
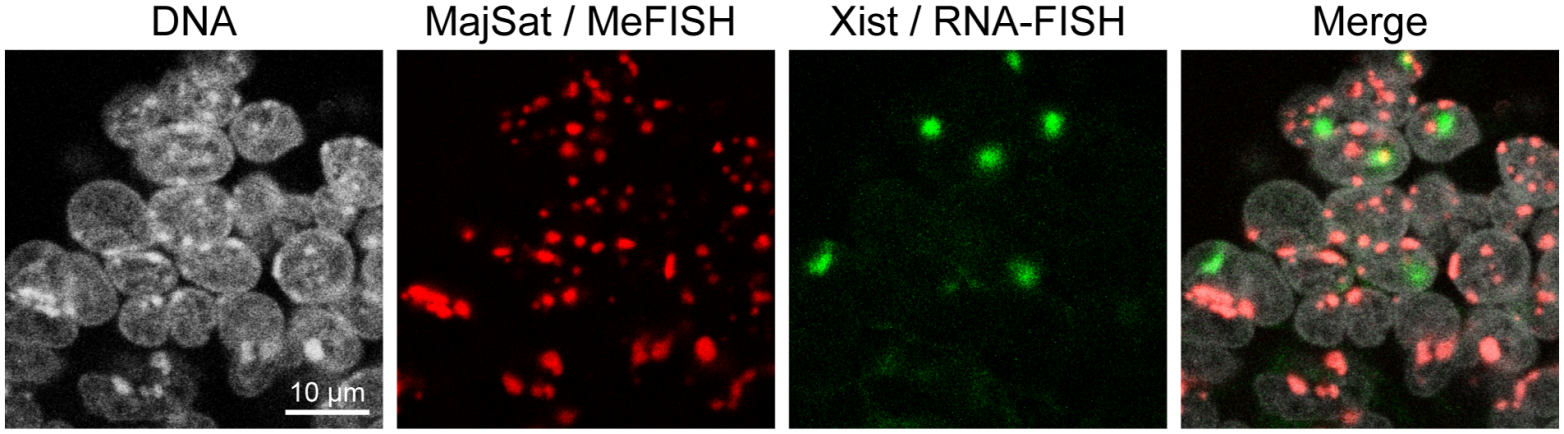
Simultaneous visualization of the DNA methylation of major satellite sequences and *Xist* expression. DNA methylation at major satellite sequences (red) and *Xist* RNA cloud (green) were visualized in the cells of a 6.5 dpc embryo using whole-mount MeFISH and RNA-FISH. Nuclear DNA was stained with TO-PRO-3 (white).

Finally, we challenged detection of DNA methylation signals and RNA expression simultaneously via a combination of the whole-mount MeFISH and RNA-FISH techniques. As RNA molecules are unstable compared with DNA, it is desirable to perform RNA-FISH before the MeFISH procedure. However, both the genomic DNA and non-cross-linked probe denaturation steps of the MeFISH protocol would dissociate the probe–target RNA hybrids generated by the RNA-FISH procedure. To overcome this problem, we used the tyramide signal amplification (TSA) technique (see Materials and Methods). TSA is one of the signal amplification techniques that are used for immunostaining and *in situ* hybridization [17]. Here, we applied this method to detect RNA-FISH signals even after both the genomic DNA and non-cross-linked probe denaturation steps of MeFISH process [18]. Tyramide, which is a phenolic compound, is converted to a short-lived, extremely reactive free radical intermediate that binds covalently to the electron-rich regions of adjacent proteins (predominantly tyrosine residues) in the presence of peroxidase activity [17], [19], [20]. As shown in Figure S2, after hybridization of biotinylated RNA-FISH probes with target RNA, streptavidin–HRP conjugates bind to biotin in the probes, and then the conjugated HRP activates fluorescent-labeled tyramide molecules, leading to covalent binding of the labeled tyramide molecules to proteins located closely to the RNA-FISH probe. In other words, the location of the probe–target RNA hybrids can be reported by the labeled tyramide molecules even after the dissociation of the probe–target RNA hybrids. In this study, we detected *Xist* RNA, which is a noncoding RNA that is essential for X chromosome inactivation [21], as well as methylation signals of the major satellite repeats. The *Xist* RNA covers the entire inactive X chromosome of female mammalian cells, forming an “*Xist* RNA cloud”, which is a hallmark of inactive X chromosomes [22]. First, we performed *Xist* RNA-FISH on 6.5 dpc mouse embryos, followed by whole-mount MeFISH, as shown in Figure 3. The RNA-FISH signals for *Xist* were detected clearly as clouds, and the MeFISH signals were also visible at the chromocenters, as observed in Figure 1. Thus, the combination of whole-mount RNA-FISH and MeFISH allowed the visualization of RNA transcription and DNA methylation on specific sequences.

## Discussion

Li et al. [7] recently reported the MeFISH method, in which an ICON probe was used to detect the methylation status of specific DNA sequences in cells/tissue sections adhered to a glass slide. Here, we presented the whole-mount MeFISH method, which can be used to visualize the DNA methylation status of individual cells at specific loci in intact embryonic structures. The whole-mount protocol has obvious advantages for understanding spatiotemporal changes in the epigenetic status during development because information on the spatial location of each cell is retained. In combination with immunostaining, this technique can delineate the DNA methylation status of target sequences in specific cell lineages within an embryo. In fact, the methylation status of the satellite repeats in rare cell populations, such as PGC precursors or developing PGCs, was successfully demonstrated in this study.

The methylation status of repeat DNA sequences has served as an important measure of the progress of epigenetic reprogramming [9]–[11]. However, methylation analysis of repeated sequences has not been performed with single-cell resolution during the DNA demethylation process in PGCs. The data obtained using 12.5 dpc PGCs in this study is essentially similar to that obtained by bisulfite sequencing analysis [11], [16], thus confirming the validity of the MeFISH method. Therefore, whole-mount MeFISH should promote the understanding of the DNA demethylation kinetics of various repeated sequences within heterogeneous populations of developing PGCs. Moreover, because three-dimensional nuclear structures within a cell can be maintained with our protocol, the relationship between DNA methylation and the nuclear positioning of repeat sequences can be analyzed [23].

DNA methylation dynamics has been studied to understand how developmental gene expression is regulated by epigenetic changes. However, there are no reports describing the relationship between the methylation of regulatory DNA sequences and gene expression with single-cell resolution, because no methods have been developed for the simultaneous detection of RNA expression and specific DNA methylation in the same cell. With this goal in mind, we combined whole-mount MeFISH and RNA-FISH, and successfully detected the methylation status of the satellite sequences and *Xist* RNA expression in the same cell within mouse embryos. To our knowledge, this is the first report of the simultaneous detection of transcribed RNAs and specific DNA methylation at the single-cell level. This was enabled by the use of the TSA method, which is normally used for signal amplification enhancement [17], [19].

When MeFISH is combined with RNA or protein expression analysis, the harsh denaturation step of the MeFISH causes the loss of signals detected by RNA-FISH or immunostaining, making it difficult to perform such combined analysis. In particular, we experienced that RNA-FISH signals were almost completely lost during the denaturation step of the MeFISH procedure (data not shown). When RNA-FISH and DNA-FISH combination is conducted in samples fixed on a slide glass, the signals of RNA-FISH performed before DNA-FISH are imaged and the xy coordinates of the images on the glass are recorded. DNA-FISH images are then matched to the RNA-FISH signals according to the position information obtained previously, thus enabling detection of RNA- and DNA-FISH signals in the same cells [24]. However, this approach cannot be applied to the whole-mount protocol because samples are not anchored to the substratum. To overcome this problem, we used the TSA method to retain the information of the location of the probe–target RNA hybrids even after the harsh denaturation steps of MeFISH. The TSA method is also compatible with combined analysis of immunostaining and MeFISH. In addition, as the TSA method was originally devised as a signal amplification technique, staining efficiency of immunostaining can be increased by 10–100-fold using this method [25], and such signal enhancements should be beneficial particularly for MeFISH.

In this study, MeFISH was performed using probes that targeted multicopy repeat sequences. As the ICON probe was originally developed to examine the CpG methylation status of any single-copy sequence using a PCR-based assay [5], the ICON-FISH probe should bind to any single-copy target sequences in a methylation-dependent manner. Thus, the limiting factor in the detection of MeFISH signals *in situ* seems to be the number of fluorophores that associate with the target DNA region. In our preliminary experiments, MeFISH signals for mouse minor satellite sequences were detected without any signal amplification (data not shown). Mouse minor satellite repeats span a 300–600 kb sequence at the centromeric region of all chromosomes other than the Y chromosome and consist of a 120 bp unit [12], [26], i.e., approximately 2,500–5,000 copies of the repeat unit exist on each mouse chromosome. Our results showed that the methylation status of these 2,500–5,000 copies can be detected without any signal amplification. Given that the TSA method can enhance signal intensities by 10–100-fold, the minimum copy number of the target sequence that can be detected by the TSA-enhanced MeFISH is 25–50, in theory. Multiple rounds of TSA may enhance signal strength further. *In situ* detection of chromosomal loci has been attempted using various fluorescent-labeling means [27]–[29], in which the detection of single-copy genomic loci has been a challenging task. Chen et al. [29] succeeded for the first time in visualizing single-copy sequences using GFP-tagged modified Cas9 proteins in human cells. In their study, multiple guide RNAs were designed from a 5 kb nonrepetitive region; 26–36 Cas9-GFP molecules recruited by different guide RNAs were sufficient to visualize the single-copy locus. By analogy, the development and usage of multiple probes from a target locus may also be effective in MeFISH. Further improvements of the MeFISH protocol, including reevaluation of ICON probe sequences and length to attain a better signal-to-noise ratio and probe penetration, development of multiple probes from target sequences, and multiple rounds of TSA amplification, should enable the detection of DNA sequences with a lower copy number and, ultimately, the detection of single-copy target sequences.

In conclusion, whole-mount MeFISH exhibited at least two advantages: 1) it provides spatiotemporal information on DNA methylation changes at specific loci and 2) theoretically, it can retrieve information on the DNA methylation of specific sequences, localization of specific proteins, and expression of specific RNA transcripts with single-cell resolution. Therefore, the whole-mount MeFISH technique should provide new opportunities for the study of the epigenetic basis of cellular dynamics during embryonic development.

## Supporting Information

Figure S1 **Overview of MeFISH.** After denaturation of genomic DNA in samples, labeled ICON probes are hybridized with target DNA. Hybridized ICON probes are cross-linked by osmium treatment when the target cytosine is methylated (or hydroxymethylated) [4]. After denaturation of ICON probes that are not cross-linked, MeFISH signals are observed using a fluorescence microscopy. Hexagons and “B” in the ICON probe indicate biotin (or fluorescence) labeling and a bipyridine-attached adenine, respectively. “C” and “5 mC” in target genomic DNA indicate unmodified and methylated cytosine, respectively. “Os” indicates osmium. A) The unmodified cytosine at the target site is not cross-linked with the MeFISH probe after osmium treatment, and the probe can be removed by denaturation. B) The methylated cytosine at the target site is cross-linked with the ICON probe when treated with osmium, leading to signal detection (depicted as a gray dot in the cellular nucleus). C) If samples are not treated with osmium, the ICON probe is removed by denaturation, even though the target sequence contains methylated cytosine.
(TIF)

Figure S2 **Overview of tyramide signal amplification (TSA) in RNA-FISH.** (1) Biotinylated probes (arrows with white circle) are hybridized with nascent target RNA (wavy lines). (2, 3) After binding of streptavidin–HRP conjugate (indicated as “HRP”) to biotin in the probes, fluorescence-labeled tyramide molecules (gray ovals with black star) are activated by the conjugated HRP. (4) Activated tyramide molecules (white ovals with black star) bind covalently to proteins that are adjacent to RNA–probe hybrids. (5) The labeled tyramides with fluorescence labeling remain as RNA-FISH signals even after dissociation of the probes during MeFISH procedures (black stars indicate fluorescence labeling).
(TIF)

Movie S1 **Movie of the optical serial sections shown in** **Figure 2B** **.**
(MOV)

Movie S2 **Movie of the 3D reconstructed images shown in** **Figure 2B** **.**
(MOV)
