# Supplementary figures and images for: Whole-Mount MeFISH: A Novel Technique for Simultaneous Visualization of Specific DNA Methylation and Protein/RNA Expression
Source: PLoS One. 2014 Apr 22;9(4):e95750. doi: 10.1371/journal.pone.0095750 (PMC3995954; doi:10.1371/journal.pone.0095750)

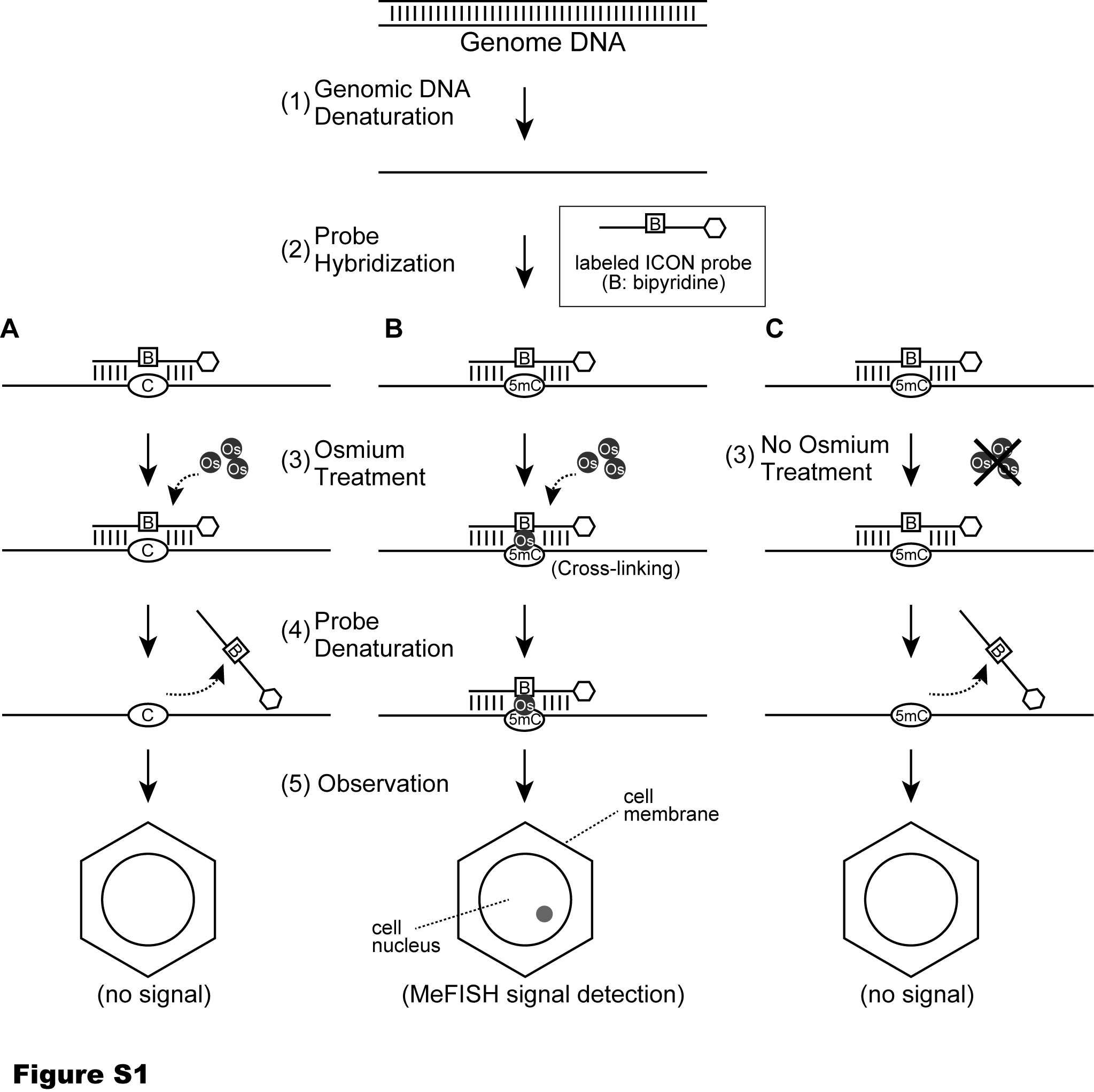

Supplement: Figure S1 — Overview of MeFISH. After denaturation of genomic DNA in samples, labeled ICON probes are hybridized with target DNA. Hybridized ICON probes are cross-linked by osmium treatment when the target cytosine is methylated (or hydroxymethylated) [4]. After denaturation of ICON probes that are not cross-linked, MeFISH signals are observed using a fluorescence microscopy. Hexagons and “B” in the ICON probe indicate biotin (or fluorescence) labeling and a bipyridine-attached adenine, respectively. “C” and “5 mC” in target genomic DNA indicate unmodified and methylated cytosine, respectively. “Os” indicates osmium. A) The unmodified cytosine at the target site is not cross-linked with the MeFISH probe after osmium treatment, and the probe can be removed by denaturation. B) The methylated cytosine at the target site is cross-linked with the ICON probe when treated with osmium, leading to signal detection (depicted as a gray dot in the cellular nucleus). C) If samples are not treated with osmium, the ICON probe is removed by denaturation, even though the target sequence contains methylated cytosine. (TIF) [file pone.0095750.s001.tif]

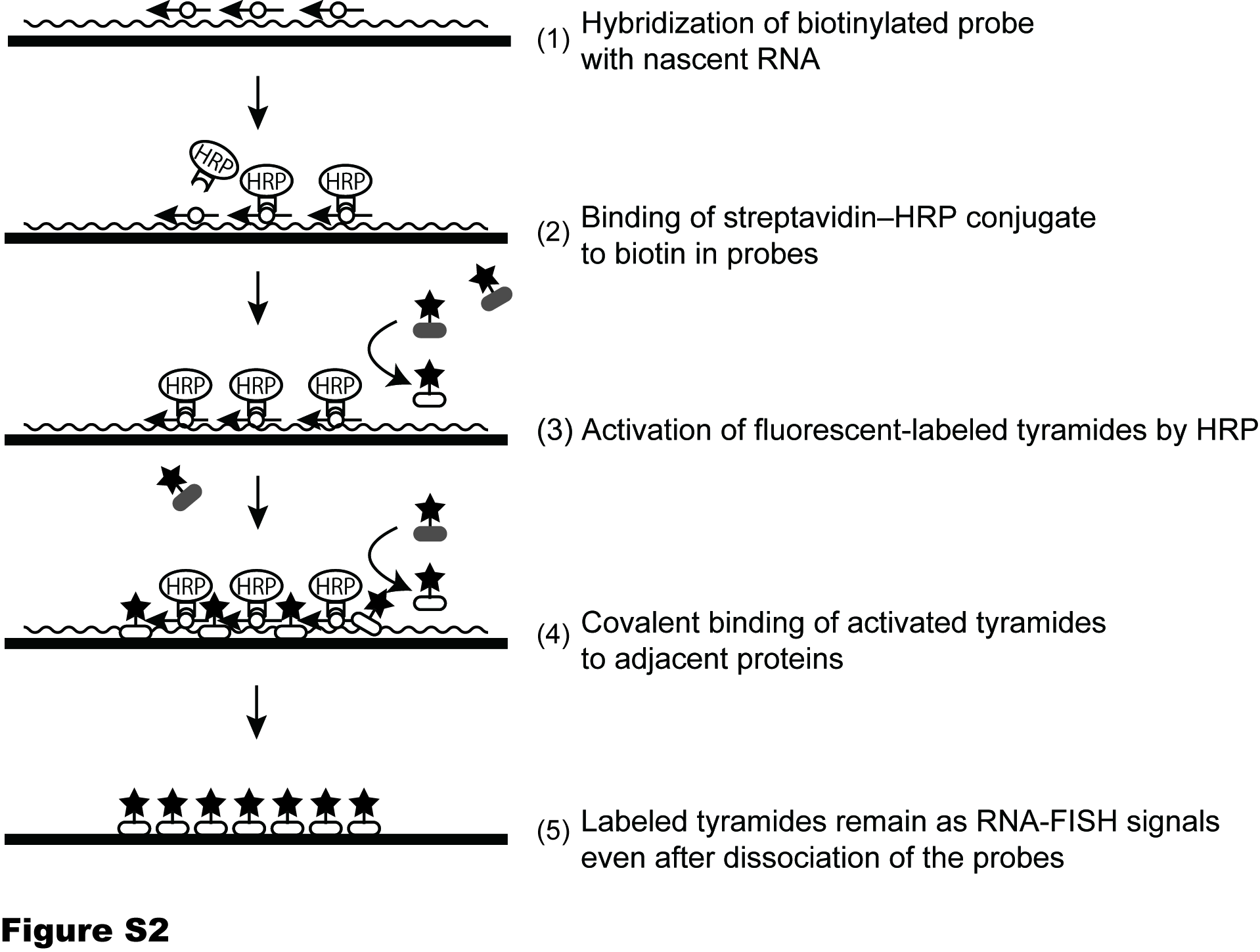

Supplement: Figure S2 — Overview of tyramide signal amplification (TSA) in RNA-FISH. (1) Biotinylated probes (arrows with white circle) are hybridized with nascent target RNA (wavy lines). (2, 3) After binding of streptavidin–HRP conjugate (indicated as “HRP”) to biotin in the probes, fluorescence-labeled tyramide molecules (gray ovals with black star) are activated by the conjugated HRP. (4) Activated tyramide molecules (white ovals with black star) bind covalently to proteins that are adjacent to RNA–probe hybrids. (5) The labeled tyramides with fluorescence labeling remain as RNA-FISH signals even after dissociation of the probes during MeFISH procedures (black stars indicate fluorescence labeling). (TIF) [file pone.0095750.s002.tif]
